# The role of audience participation and task relevance on change detection during a card trick

**DOI:** 10.3389/fpsyg.2015.00013

**Published:** 2015-02-05

**Authors:** Tim J. Smith

**Affiliations:** Department of Psychological Sciences, Birkbeck, University of London, London, UK

**Keywords:** card trick, change blindness, attention, perception, agency, web experiment, magic

## Abstract

Magicians utilize many techniques for misdirecting audience attention away from the secret sleight of a trick. One technique is to ask an audience member to participate in a trick either physically by asking them to choose a card or cognitively by having them keep track of a card. While such audience participation is an established part of most magic the cognitive mechanisms by which it operates are unknown. Failure to detect changes to objects while passively viewing magic tricks has been shown to be conditional on the changing feature being irrelevant to the current task. How *change blindness* operates during interactive tasks is unclear but preliminary evidence suggests that relevance of the changing feature may also play a role (Triesch et al., 2003). The present study created a simple on-line card trick inspired by Triesch et al.’s (2003) that allowed playing cards to be instantaneously replaced without distraction or occlusion as participants were either actively sorting the cards (*Doing* condition) or watching another person perform the task (*Watching* conditions). Participants were given one of three sets of instructions. The relevance of the card color to the task increased across the three instructions. During half of the trials a card changed color (but retained its number) as it was moving to the stack. Participants were instructed to immediately report such changes. Analysis of the probability of reporting a change revealed that actively performing the sorting task led to more missed changes than passively watching the same task but only when the changing feature was irrelevant to the sorting task. If the feature was relevant during either the pick-up or put-down action change detection was as good as during the watching block. These results confirm the ability of audience participation to create subtle dynamics of attention and perception during a magic trick and hide otherwise striking changes at the center of attention.

## INTRODUCTION

Our perception of the visual world is fallible. We may believe we have direct access to a rich and reliable mental representation of our visual environment but evidence from studies in which features of the scene have been unexpectedly changed have shown that we are remarkably unaware of such changes when they are hidden during a period of distraction such as a flicker, an eye blink, or saccadic eye movement (*change blindness*; Simons and Rensink, 2005). Such experimental techniques for exposing change blindness are relatively recent but magicians and pickpockets have been exploiting these limitations for millennia. Magicians commonly refer to such manipulation of awareness as *misdirection*: any technique used to direct audience attention away from the method by which the magician creates the effect (Lamont and Wiseman, 1999; Kuhn and Martinez, 2011). For example, a magician’s glance at his right hand (the misdirection) may be used to draw attention away from his left hand as it drops a cigarette lighter in his lap (the method) and then reveals its magical disappearance (the effect; Kuhn et al., 2008).

Misdirection takes many forms and has been categorized in many ways by both magic theorists and, more recently psychologists (see Kuhn and Martinez, 2011, for review). For example, Sharpe (1988) distinguished two types of misdirection: active and passive. *Active* misdirection involves the movement of spatial attention via some transient change in sound or movement. *Passive* misdirection is described as the misdirection of the mind by influencing how audience members see or react to the stimuli they are attending to (as quoted in Kuhn and Martinez, 2011). This distinction seems useful for characterizing the techniques magicians use for misdirection but does not provide sufficient detail for the psychological components of misdirection to be identified or investigated. A recent psychological taxonomy of misdirection (published in this special issue; Kuhn et al., 2014) addresses such limitations by casting misdirection in terms of psychological theories of perception (including attention), memory and reasoning. Kuhn et al. (2014) pointed out that classic theories of misdirection, such as Sharpe (1988) and Lamont and Wiseman (1999) often emphasize the role manipulating attention plays in creating the misdirection but fail to distinguish between the locus of control of attention (*exogenous vs. endogenous*) or what form attention takes (*overt vs. covert*). Sharpe’s (1988) active/passive distinction is somewhat similar to the psychological distinction between *exogenous* control (involuntary control of attention by external sensory events) and *endogenous* control (voluntary control of attention by cognitive factors such as preference, task or understanding) but it also conflates *overt* attention (the physical movement of the sensory apparatus to point at an attended target, e.g., an eye movement) and *covert* attentional shifts (the reallocation of processing resources either away from the point of overt attention or to different features at fixation; Rensink, 2013). Sharpe’s (1988) categories also suffer from using intuitive terminology that bear the weight of colloquial interpretations. *Active* typically refers to behaviors that are effortfully engaged in, whereas *passive* is the opposite, i.e., a lack of active behavior. In the context of magic tricks these common meanings may more intuitively be used to distinguish between tricks that involve audience participation (active) vs. tricks in which the audience is simply watching it unfold. These more intuitive meanings will be used in the present study.

In order to look for empirical evidence of how these psychological processes (exogenous vs. endogenous control; overt vs. covert attention; and active vs. passive participation) are used in misdirection we can first identify their role in the related and more comprehensively studied phenomena, change blindness. Evidence for misdirection of *overt* attention as a method for inducing change blindness is common (Simons and Rensink, 2005). For example, change blindness is greater for objects away from areas of central interest in a photograph when changes occur across flickers (Rensink et al., 1997), is created by non-occluding “mudsplashes” that involuntarily attract attention (O’Regan et al., 1999) and increases with distance from fixation when the change occurs across a saccade (Henderson and Hollingworth, 1999). The impact of fixation location on change blindness has also been clearly demonstrated in a specially designed card trick (Smith et al., 2013). In this trick a deck of blue-backed cards was switched for a deck of red-backed cards in full sight (i.e., without occlusion or distraction) but participants failed to notice as their eyes were fixated on a different location as the cards were dealt.

Evidence for misdirection of *covert* attention is less clear. In the aforementioned card trick (Smith et al., 2013), exogenous cues (e.g., a flashing ring around the card backs) were used to try and attract overt attention back to the site of the change but even when a few participants fixated the card backs as they changed color nobody identified the change. This suggested a dissociation between overt and covert attention at fixation, a property of visual attention first identified by von Helmholtz (1896). Similar evidence of this dissociation has been shown when the change occurs across an eyeblink and participants fail to detect the change even when they are fixating it before and after the blink (O’Regan et al., 2000). Failure to detect a dropped object during a magic trick has also been repeatedly shown to be independent of fixation location and therefore, overt attention (Kuhn and Tatler, 2005; Kuhn et al., 2008; Kuhn and Findlay, 2010). This effect suggests that either covert attention has shifted away from fixation or is prioritizing features at fixation that are not indicative of the critical feature. This latter case could be considered an example of *contingent capture* (Folk et al., 1992). Deployment of attention is dependent on “attentional control settings” and a feature may not capture attention unless it shares the same feature as the target, such as color (Folk and Remington, 1998). The influence of feature relevance on change detection has also been demonstrated in simple letter arrays (Cole et al., 2009).

Clear evidence of covert misdirection at fixation has been provided by Smith et al. (2012) using a coin trick. Participants failed to notice a change in identity of a coin even though they were attending to and fixating it as it was changed during a very brief occlusion by the magician’s hand. Participants were instructed to guess whether the coin would land heads or tails up when it was dropped after an unknown number of passes between the magician’s hands. Prioritizing the face of the coin de-emphasized the monetary value and identity of the coin even though both sets of visual features were coincident at fixation. The design of this trick ensured attention remained at fixation throughout the trick but this did not guarantee change detection as the feature that changed was not relevant to the viewing task.

If the aforementioned coin trick demonstrated how changes to a visual feature at fixation may not be detected when the visual feature is unrelated to the viewing task then increasing feature relevance should increase detection. The impact of viewing task (i.e., endogenous control) on change detection at fixation was demonstrated by Triesch et al. (2003) in a pivotal study that used Virtual Reality to make instantaneous changes to objects whilst they were being manipulated by participants. In this study, participants were instructed to sort virtual blocks on to two conveyor belts according to one of three instructions: (1) “Pick up the bricks in front to back order and place them on the closer conveyor belt.” In this case block size was irrelevant during both the pick-up and placement of each block; (2) “Pick up the tall bricks first and put them on the closer conveyor belt. Then, pick up the small bricks and also put them on the closer conveyor belt.” For this condition size only mattered during block pick-up; (3) “Pick up the tall bricks first and put them on the closer conveyor belt. Then, pick up the small bricks and put them on the distant conveyor belt.” For this instruction block size was relevant for both the pick-up and placement action. As participants picked-up a block and moved it to the conveyor belt the size of the block occasionally changed. The frequency with which participants spontaneously reported these changes increased with the task relevance of block height (Instruction 1 < 2 < 3) with the majority of participants (88%) not reporting any changes with the first set of instructions. Analysis of eye movements indicated that most changes happened during or immediately before or after a saccade which may indicate that saccadic suppression helped obscure the transients associated with the size change. However, even if the block was being tracked by the eyes during the change this did not guarantee change detection. These results indicated that the relevance of an object feature to the task at a particular moment influences whether that feature will be encoded and available for change detection. The authors hypothesized that information was extracted “just in time” to solve the current goals (Triesch et al., 2003).

Similar evidence of the impact of “just in time” relevance on change detection at fixation is difficult to find and a replication of the Triesch et al. (2003) findings has not been forthcoming (except for by the same group using a similar setup; Droll et al., 2005). The main difficulty in replicating these findings is the complex VR setup used to induce the changes during an interactive task without distraction (e.g., flicker, blink, or occlusion). Instantaneous transformation or replacement of an object is physically impossible in real-life or even during a magic trick. All “magical” transformations will either involve active misdirection of attention away from an object during the change or momentary occlusion (as in Smith et al., 2012). If such distractions are to be avoided a virtual environment must be used.

The closest evidence of task relevant change detection during an active task comes from a study using a driving simulator (Wallis and Bülthoff, 2000). In this study, participants were instructed to explicitly detect changes to blocks positioned by the side of a road as they either actively steered the virtual car down the road, watched a video of the same motion or looked at a static slideshow of the same path. All changes were obscured with a brief flicker. Wallis and Bülthoff (2000) found that change detection increased as the location of the blocks neared the driving line but only when the participant was actively steering the car around the blocks. When the same scene was presented as a passive video or static slideshow, proximity of the blocks to the driving line did not have an effect on change detection and overall change detection was greater than in the active viewing condition. Whether the task difference was due to relevance, e.g., the blocks in the road had to be negotiated in the active condition, or proximity to attentional focus, e.g., in the active condition attention must be focussed on the road whereas attention was free to explore the passive and static scenes, cannot be known as the location of viewer attention was not controlled during this study. However, the counter-intuitive finding that change detection was worse during an active task than a passive task is intriguing and raises the question of whether Triesch et al.’s (2003) findings are a consequence of how attention is allocated during a physically active task or whether task relevance would also impact change detection in a similar but passively viewed task.

Support for the use of an active task to limit viewer awareness can be found in the magic literature.

“Whenever possible in routining a trick, make use of as many persons from the audience as possible. The use of a committee not only makes amusing by-play possible, but it affords excellent cover for secret sleights.… by having a committeeman provide the misdirectional cover you need for the secret sleight”

(Hugard and Braue, 1944, p. 446).

The misdirectional cover Hugard and Braue (1944) suggest is often physical, such as switching a card behind the back of a volunteer but they also highlight the increase in drama and suspense created by actively involving volunteers. By being physically involved the volunteer believes they make the trick more difficult to pull off as they are better able to visually interrogate the magician’s actions. Empirical evidence for the impact of social presence on change detection comes from studies which have compared misdirection in magic tricks performed live compared to on video (Kuhn and Tatler, 2005; Kuhn et al., 2008). Whilst misdirection worked in both settings, it was more effective face-to-face and gaze behavior or detection rates were not changed by viewing instructions. This evidence is supported by a growing literature demonstrating that the social presence of another person and the potential for interaction (i.e., not presented via a video screen or one-way mirror) alters viewer gaze behavior (Risko et al., 2012).

Hugard and Braue (1944) also suggest that actively involving a volunteer in a magic trick provides another opportunity for misdirection. The volunteer will focus intently on the given task such as shuffling the cards and, in doing so, fail to attend to seemingly irrelevant elements that are critical for the magician’s success such as the removal of a card from the deck (Hugard and Braue, 1944). This intuition mirrors recent empirical findings. When actively engaging in a physical task visual attention is focussed on task relevant objects that are about to be picked up or are currently being manipulated (Land et al., 1999; Hayhoe et al., 2003). The distribution of fixations within an interactive task varies depending on what task is being performed (Rothkopf et al., 2007) and is more focussed on task relevant objects during the task than before starting the task (Hayhoe et al., 2003). Such task-specific momentary influences on attention may explain change blindness demonstrations in real-world scenes such as a failure to detect a change in the identity of a conversational partner when giving directions on a map (Simons and Levin, 1998). By actively engaging a participant in a viewing task the magician may be increasing the predictability of how attention is allocated over time and provide opportunities for their manipulations to pass unseen.

The present study set out to investigate whether task relevance would influence change detection at fixation during a passive task in the same way it has been previously demonstrated during an active task (Triesch et al., 2003). To provide the empirical control required and the ability to instantaneously change features of an object during manipulation without the need for a distractor (e.g., a flicker or occlusion) a novel on-line card task was devised. The card task involved participants sorting playing cards on to two piles (known as “stacks”) according to instructions. The instructions varied in the degree to which the color of the cards was relevant to the sorting task (similar to the block size manipulation of Triesch et al., 2003). Participants were either instructed to sort the cards themselves (*Doing* condition) or watch another participant perform the sorting and check whether they followed the instructions correctly (*Watching* condition). By asking participants to judge the correctness of the card moves during the watching task the allocation of viewer attention over time should be similar to during the doing task and can be assumed without the need for eye tracking (which was not possible given the on-line nature of this study). Whilst the cards were moved from their starting positions to the stack, the color of a card would occasionally change (whilst maintaining its value, e.g., a nine of clubs would change to a nine of hearts). Participants were instructed to report a change as soon as it was detected. Given previous findings (Wallis and Bülthoff, 2000; Triesch et al., 2003; Smith et al., 2012) the momentary relevance of the card color to the sorting task was predicted to increase change detection and this effect would interact with whether the participants were actively performing the task or passively watching it, with a greater effect of instruction predicted in doing rather than passive viewing. Replication of the earlier effects using a simpler on-line task would also provide a method for future investigation of the dynamics of attention allocation during interactive tasks and its influence on object and scene representation.

## MATERIALS AND METHODS

### PARTICIPANTS

Participants were recruited on-line via the Birkbeck/UCL SONA experimental participant portal or by personal invitation by the experimenter. Fifty-seven participants completed the experiment but of these only 42 met the inclusion criteria stated below (mean age = 29.26, age range = 18–64, female = 31). Participants were excluded from analysis if timings between trials were irregular, participants did not complete the doing task or respond to the watching task correctly on the majority of trials or the experiment ended before all trials were completed.

### DESIGN

**FIGURE 1:**
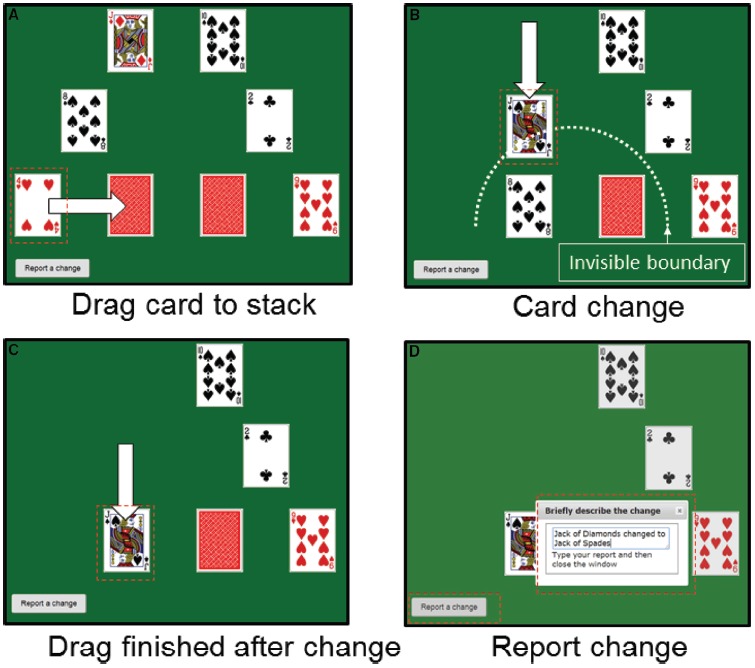
**Example frames from the card sorting task.** Participants were presented with six playing cards arranged in a semi-circle around two card stacks (i.e., face down cards). Their task was to move the cards in a specified order on to the stacks (Doing task) or watch somebody else complete the task and comment if they followed the instructions correctly (Watching task). If they notice a card change they described the change by clicking on “Report a change.” **(A)** A participant drags the four of hearts to the left stack; **(B)** the Jack of Diamonds changes to a Jack of Spades as it is dragged across the invisible boundary (dotted line); **(C)** the Jack is dropped on the left stack; **(D)** the change is reported. The task continued after the reporting window had been closed.

Participants took part in a card sorting game on-line. They were presented with 40 trials in which six playing cards were presented face-up in a semi-circle around two stacks (face-down cards with red-backs). See Figure 1A for layout of the display. Trials were divided into two blocks, 20 trials each. In one block participants were instructed to sort the cards onto the stacks in a specified order by dragging them with the mouse. This was the *doing* block. In the other block they were told to watch another participant (actually a computer simulation) complete the task according to the same rules and judge at the end of each trial if they completed the task correctly by clicking Yes/No. This was the *watching* block. Block order was counterbalanced across participants. There were three instruction conditions (1) pick up cards left to right and place on left stack (=color irrelevant); (2) pick up red cards and place on left stack then pick up black cards and place on left stack (=color only relevant during pick-up); (3) pick up red cards and place on left stack then pick up black cards and place on right stack (=color relevant during pick-up and placement). Instructions varied across participants but were the same across both doing and watching blocks for each participant. Therefore, the design was 2 (*Task*; Within subjects) × 3 (*Instruction*; Between subjects) mixed design.

While participants were completing the task they were informed that cards may occasionally change their “*number and/or suit.*” If they noticed a change they should “*click the ‘Report a Change’ button as quickly as possible. Include brief details in the pop-up, e.g., seven clubs changed. If you can’t remember what changed just write ‘don’t know’*.” The trial continued after they closed the response window (see Figure 1D). There were 10 changes per block with a maximum of one per trial. The order of trials was randomized across participants.

Text responses along with when they were made were recorded in the results. The accuracy of each reported change was checked but only recorded as a miss if they reported a change to the incorrect card or before the change happened. The order in which cards were dragged and which stack they were dragged to was also logged during the Doing trials. In the Watching task, the movement of the cards was simulated by animating card dragging using a similar pattern and speed to actual human performance. Fifty percent of trials were incorrect in the Watching task and each error involved a single card being placed on the wrong stack. Participants assessed whether each trial had correctly followed the instructions and responded *Yes/No* after each trial. These responses along with any change detection reports were logged for each Watching trial.

Analysis was performed based on the proportion of total changes (maximum 10 per block) correctly reported by participants. The number of false alarms was negligible so is ignored in subsequent analyses.

### STIMULI AND APPARATUS

The stimuli used were 2D bitmap images of the Standard (i.e., French) 52 card playing card deck (see Figure 1). All cards from the deck were used across the study including the Royal and Ace cards (but not Jokers). When a card changed color it involved an instantaneous replacement of one bitmap image with another. The change occurred across one screen refresh, the rate of which varied according to each participant’s display. The change in color was accomplished by flipping the card’s suit whilst keeping the number the same, e.g., seven of hearts to seven of spades.

The study was conducted on-line to ensure maximum participant recruitment. The experiment ran in the web browser and before starting the experiment participants were instructed to close other programs, minimize distractions in their immediate environment and maximize the browser window. JavaScript was used to code the experiment, record participant responses, mouse clicks and card moves.

As the participant completed the study their data was uploaded into a MySQL database which was immediately accessible to the experimenter via a web interface. Data were exported into a CSV file for analysis.

## RESULTS

The proportion of changes reported correctly by participants (out of 10) was calculated for each viewing task (Watching vs. Doing) and instruction condition. A mixed ANOVA with factors Task and Instruction on the proportion of changes detected per participant revealed no significant main effects of Task [*F*(1,39) = 0.842, *p* = 0.364], or Instruction [*F*(2,39) = 1.305, *p* = 0.283] but a significant interaction Task × Instructions [*F*(2,39) = 3.775, *p* = 0.032, ${\text{η}}_{\text{p}}^{\text{2}}$ = 0.162].

**FIGURE 2:**
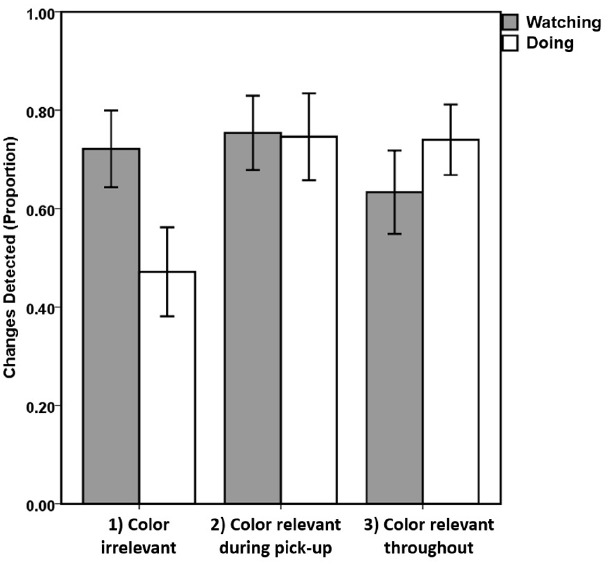
**Mean proportion of changes detected.** Bars represent each task (Doing = clear bars; Watching = solid grey bars) and instruction conditions (1 = sort cards left to right/color irrelevant; 2 = sort red on left then black on left/color relevant during pick-up; 3 = sort red to left then black to right/color relevant throughout task). Error bars represent +/–1 standard errors about the individual means.

Figure 2 clearly demonstrates the Task × Instruction interaction. The changes detected within the Watching task do not change across Instruction conditions [*F*(2,41) = 0.616, *p* = 0.545] with all three means being very similar: Instruction 1 = 0.721 (SD = 0.29), Instruction 2 = 0.754 (SD = 0.27), Instruction 3 = 0.633 (SD = 0.32). Instruction 3 detection is numerically lower than 1 and 2 but not statistically (both *t*s < 1).

By comparison, within the Doing task the main effect was significant [*F*(2,41) = 3.552, *p* = 0.038, ${\text{η}}_{\text{p}}^{\text{2}}$ = 0.154] due to Instruction 1 producing fewer detections (mean = 0.472, SD = 0.338) than Instruction 2 [mean = 0.746, SD = 0.317; *t*(25) = 2.170, *p* = 0.04 uncorrected; *p* = 0.08 Bonferroni–Hochberg corrected] and Instruction 3 [mean = 0.74, SD = 0.277; *t*(27) = 2.345, *p* = 0.027 uncorrected; *p* = 0.08 corrected]. Bonferroni–Hochberg correction was used for all multiple comparisons as this is less likely to result in false negatives than traditional Bonferroni correction whilst still retaining the familywise error at 5%, i.e., 95% confidence that the null hypothesis is correctly rejected. There was no difference between 2 and 3 [*t*(26) = 0.055, *p* = 0.957 uncorrected; *p* = 1.0 corrected]. Paired comparisons between detection for Doing and Watching only showed a significant difference within Instruction 1 [Watching > Doing; *t*(13) = 3.381, *p* = 0.005 uncorrected; *p* = 0.015 corrected] and not for 2 [*t*(12) = 0.086, *p* = 0.93 uncorrected/corrected] or 3 [*t*(14) = –0.946, *p* = 0.36 uncorrected; *p* = 0.720 corrected].

**FIGURE 3:**
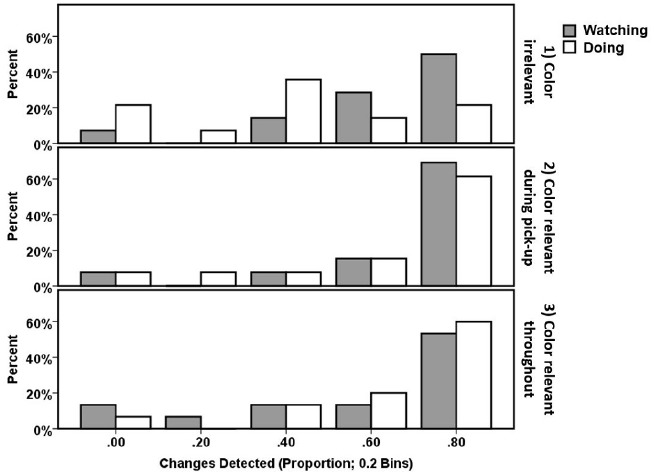
**Histogram showing the percentage of participants in each Instruction condition that had a particular proportion of change detection (divided into five bins: 0–0.19, 0.2–0.39, 0.4–0.59, 0.6–0.79, 0.8–1.0).** Bar colors indicate the Task block (gray = Watching; white = Doing).

The analysis above demonstrated that when the instructions were simple and the feature that changed (i.e., the color) was irrelevant to the task participants reported less changes but only when they are actively performing the task. When participants were passively watching the task and assessing if the instructions were followed correctly the instructions had no impact on change detection. This interaction resulted in the rather counter-intuitive better detection in *Watching* than *Doing* for Instruction 1. This change in detection across instruction conditions can also be seen in the distribution of participants who produced particular detection rates (Figure 3). For all conditions other than Doing + Instruction 1, the modal detection proportion was 0.8–1.0. For Doing + Instruction 1 the mode shifted to 0.4 and there was also an increase in the number of participants failing to detect any changes, 21.4% compared to ∼7% for all other conditions (except for 13.3% Watching + Instruction 3). This distribution of detection rates indicates that even in the condition with the worst average detection rate (Doing + Instruction 1) change detection for some participants within this group was very good, whereas other participants were poor. This suggests that the lower cognitive demands of Instruction 1 may have led to some participants paying less attention to the cards and, as a result detecting fewer changes. By comparison, the higher demands of Instructions 2 and 3 gave less opportunity for inattention if participants were to complete the task correctly. However, there is no evidence that participants in Instruction 1 were allocating an insufficient level of attention to the card sorting task as their identification of whether the task was performed correctly during the watching condition (mean accuracy = 0.96, SD = 0.09) was as good as under all other instructions [Instruction 2: accuracy = 0.93, SD = 0.13; Instruction 3: accuracy = 0.98, SD = 0.056; *F*(36) = 1.154, *p* = 0.327]. The key difference appears to be the visual features to which attention was allocated, not the overall level of attention.

## DISCUSSION

The results presented here confirm the intuition of magicians that asking an audience member to actively participate in a trick provides greater opportunity for misdirection at fixation than passively watching the trick. Watching the card sorting and judging whether it was performed correctly did not lead to the same changes in sensitivity to task-related visual features as performing the task. Participants missed more color changes when they were sorting the cards but only when the color of the cards was irrelevant to the task (i.e., Instruction 1). This difference between doing and watching mirrors that found by Wallis and Bülthoff (2000). In their driving simulator study, participants were worse at detecting changes to blocks in the dynamic scene when they were actively steering the car compared to watching a video of a similar scene (Wallis and Bülthoff, 2000). This effect interacted with the location of blocks relative to the driving line: changes to blocks closer to the driving line were detected more than those further away. However, given that the active task was to navigate blocks on the road it was unclear whether this location effect was due to the irrelevance of the distant blocks to the task or their distance from the likely focus of attention, i.e., the road. In the present study, participant attention had to be allocated to each card as it was selected, dragged, and placed precisely on the stack. This pattern of attention should not have altered across instruction conditions even though which cards were selected and where they were placed changed. As such, the observed effect of instruction on change detection can be attributed to differences in the processing of information at the center of attention rather than differences in the location of attention. However, slight differences in eye movements may have occurred across instruction conditions such as more anticipatory saccades to the next card in the simpler Instruction 1 compared to the other instruction conditions. Although earlier studies have suggested that fixation location does not influence change detection during such dynamic scenes (Triesch et al., 2003; Kuhn and Tatler, 2005; Kuhn et al., 2008; Kuhn and Findlay, 2010; Smith et al., 2012, 2013) we cannot rule out the possibility that subtle eye movement differences may have dissociated attention from the critical card as it changed, providing an opportunity for change blindness. Future studies should monitor eye movements during this interactive task to discount this possibility.

The observed relationship between change blindness and the task relevance of the changing visual feature confirms previous findings during active tasks (Wallis and Bülthoff, 2000; Triesch et al., 2003; Smith et al., 2012). However, whereas Triesch et al. (2003) demonstrated an increase in change detection when the critical feature was relevant throughout the task compared to just during object pick-up we found no difference between these conditions, i.e., Instructions 2 and 3. Our active results (i.e., the *Doing* condition) suggest that change detection is only impaired when the critical feature is completely irrelevant to the task rather than the “just in time” relevance previously argued for (Triesch et al., 2003). However, even in the earlier study it is unclear how “just in time” processing explains their findings. The block change always occurred mid-way between the pick-up and put-down areas (as in the present study) which meant that even in their Instruction 2 (a parallel to ours) the critical feature was no longer relevant to the task as the object has already been selected based on that feature. The up-coming object placement decision did not require maintenance of the critical object feature suggesting that if only visual information immediately relevant to the task was extracted from the attended object there should have been no change detection. Their evidence of a moderate amount of change detection in Instruction 2 suggests that either the previously relevant feature is still maintained in working memory even after relevance (permitting the correspondence between the current feature and that held in memory; Simons, 2000) or the prior relevance of the feature creates some residual attentional presetting (Folk et al., 1992) allowing the change to capture attention. It is, however worth noting that not all “just in time” theories imply that attention is immediately removed from an object or feature after it has ceased being relevant (Rensink, 2000). Such a theory would accommodate the results presented here or in Triesch et al. (2003).

In order to further explore the time course of feature relevance, Droll et al. (2005) modified their earlier VR block sorting task (Triesch et al., 2003) and instructed participants to use different visual features for the pick-up and put-down actions. Irrespective of whether the changing feature had been relevant in the recent past (during pick-up) or was going to be relevant in the near future (during put-down) explicit change detection was the same and significantly greater than changes to irrelevant features. They interpreted these findings as indicating that once a feature is used in a subtask it is not immediately discarded from working memory. Similarly, features are encoded and maintained in working memory before they are strictly required (e.g., for the placement decision). This pattern of a prolonged influence of task relevance on visual encoding and maintenance in working memory also fits our evidence of greater change detection in both Instruction 2 and 3. Visual features are not encoded by default when an object is attended (as suggested by *object file* theory; Kahneman et al., 1992) nor are the encoded features restricted only to those that are immediately relevant (Triesch et al., 2003). Instead, relevance seems to have a longer time course which is probably dictated by the event structure and cognitive demands of the task. Future research should investigate how prolonged the relevance effect is on working memory maintenance and how it interacts with working memory capacity.

Our findings extend recent evidence of the modulation of attention and feature encoding during the passive viewing of dynamic scenes (Zacks et al., 2001; Levin and Varakin, 2004; Smith and Henderson, 2008; Smith and Martin-Portugues Santacreu, under review) and active visuomotor tasks (Hayhoe et al., 1998; Baldauf and Deubel, 2010). Whilst watching videos of naturalistic scenes (Smith and Mital, 2013), human event sequences (Zacks et al., 2001), and edited films (Levin and Varakin, 2004; Smith and Henderson, 2008; Smith and Martin-Portugues Santacreu, under review) the availability of visual attention appears to fluctuate over time (Levin and Saylor, 2008) along with the dynamic low-level and semantic features of the depicted scenes. These changes provide opportunities for large visual disruptions such as blank frames (Levin and Varakin, 2004) or cuts between viewpoints (Smith and Henderson, 2008; Smith and Martin-Portugues Santacreu, under review) to pass unnoticed. The spatiotemporal modulation of attention appears to be even more pronounced during manual activities (Baldauf and Deubel, 2010). Attention is highly focused on task relevant objects (Hayhoe et al., 2003) and spatially allocated in parallel to all movement-relevant locations before execution (Baldauf et al., 2006). However, visual target discrimination at fixation has been shown to be impaired during a grasping movement toward the fixated object (Hesse et al., 2012) or an adjacent but non-fixated object (Hesse and Deubel, 2011). This decrease in visual discrimination has been interpreted as evidence that visual attention is required for the effective control of fine hand kinematics and must be diverted from processing of visual features that are not immediately relevant to the motor action (Hesse and Deubel, 2011). The impaired change detection during doing Instruction 1 in the current study may be further evidence of this withdrawal of attention from visual feature processing and reallocation to the motor action. If this is the case it is evidence that the effect transfers through an interface device (in this instance, a computer mouse or trackpad) as the action space in which the participants moved their hands (e.g., physical desktop) and the visual space in which these actions took effect (e.g., the computer display) were spatially separated (for similar evidence using an interactive computer game see Hayhoe et al., 1998). However, by making the card color relevant to the motor action in Instructions 2 and 3 we appear to have spared such withdrawal.

Our findings suggest that actively involving participants in a manual task such as sorting cards can function as a method for misdirecting attention away from a manipulation even when it occurs at fixation. Instructing participants to passively watch the same action does not create change blindness. As such, our results confirm the intuitions of magicians for the power of audience participation (Hugard and Braue, 1944) and the potential for covert misdirection of attention at fixation by manipulating task relevance (Sharpe, 1988). However, our study also highlights the need for more nuanced psychological theories of misdirection than are usually provided by magic theorists (see Kuhn et al., 2014). For example, the absence of change blindness when the changing feature became task relevant (irrespective of when during the task it was relevant) suggests that great care must be taken to use a task which is plausible but does not require the processing of features relevant to the intended manipulation. Of course, the task and manipulation used in the present study are far removed from those typically used in a card trick and our task lacks an “effect,” such as revealing that the card a participant was dragging had changed color without them noticing. That said, our results demonstrate that even without the multiple levels of misdirection, social presence and performance typically employed by a close-up magician during a trick we were able to use the natural dynamics of visual attention during an active task to limit awareness of an impossible change at fixation. This provides further evidence of the complex dynamics of visual attention during naturalistic interactive tasks.

### Conflict of Interest Statement

The author declares that the research was conducted in the absence of any commercial or financial relationships that could be construed as a potential conflict of interest.
